# Advances in urinary biomarkers for endometrial cancer detection

**DOI:** 10.1038/s41416-026-03579-8

**Published:** 2026-08-11

**Authors:** Molly Dore, Jiexin Cao, Holly Baker-Rand, Ananya Choudhury, Anthony D. Whetton, Richard D. Unwin, Emma J. Davidson, Kelechi Njoku

**Affiliations:** 1 Department of Obstetrics and Gynaecology, St Mary’s Hospital, Manchester University NHS Foundation Trust, Manchester Academic Health Science Centre, Manchester, UK; 2 Division of Cancer Sciences, Faculty of Biology, Medicine and Health, School of Medical Sciences, University of Manchester, Manchester, UK; 3 Veterinary Health Innovation Engine, Schools of Veterinary Medicine and Biosciences, Faculty of Health and Medical Sciences, University of Surrey, Guildford, UK; 4 Centre for Reproductive Health, Institute for Regeneration and Repair, University of Edinburgh, Edinburgh, UK; 5 CRUK Scotland Centre, Institute of Genetics and Cancer, University of Edinburgh, Edinburgh, UK; 6 Centre for Evidence-Based Health Care, Nuffield Department of Primary Care Health Sciences, University of Oxford, Oxford, UK; 7 Department of Clinical Oncology, Christie NHS Foundation Trust, Manchester, UK

**Keywords:** Endometrial cancer, Molecular biology

## Abstract

The incidence of endometrial cancer is rising globally, placing significant burden on diagnostic pathways for women with abnormal uterine bleeding. Current evaluation relies primarily on transvaginal ultrasound followed by invasive endometrial visualisation and sampling, although most symptomatic women have no sinister underlying pathology. Urine offers a promising non-invasive and easily repeatable biofluid capable of capturing tumour-associated signals through both systemic renal filtration and locally shed uterine material. This review summarises emerging evidence on urinary biomarkers for endometrial cancer detection, including somatic mutations, DNA methylation markers, proteins and peptides, vibrational spectroscopy signatures, microRNAs, metabolites and urine cytology. These biomarkers collectively reflect key biological processes driving endometrial carcinogenesis, including hormonal and metabolic dysregulation, immune activation, epithelial–stromal disruption, cellular proliferation and genomic instability. Among current candidates, DNA methylation panels and mutation-based assays have demonstrated encouraging diagnostic performance, with studies reporting sensitivities and specificities exceeding 80–90% in symptomatic populations. Urine cytology also shows strong diagnostic potential in selected cohorts, while metabolomic, proteomic and spectroscopy-based approaches remain promising but require further standardisation and prospective validation. Although not yet suitable for population screening, urinary biomarkers show considerable potential for risk stratification and triage of symptomatic women, potentially reducing unnecessary invasive investigations. Clinical translation will require standardised protocols, multi-centre validation and integration into existing diagnostic pathways.

## Introduction

Endometrial cancer is the sixth most common malignancy in women worldwide, with over 420,000 new cases and approximately 98,000 deaths reported in 2022 [1, 2]. The global age-standardised incidence rate is estimated at 8.4 per 100,000 women, although substantial geographic variation exists [1, 3]. In the UK, the incidence of endometrial cancer has risen markedly over the past three decades. Cases increased by 59% between 1993 and 2018 and currently more than 10,000 cases are diagnosed annually accounting for approximately 5% of all female cancer diagnoses [4].

The rising incidence of endometrial cancer parallels demographic ageing and the escalating prevalence of obesity. Approximately 30% of the adult population in the UK are living with obesity and this is expected to rise to 55% by 2050. Obesity-related excess oestrogen, hyperinsulinaemia and chronic inflammation promote endometrial proliferation, increased cell turnover and malignant transformation and is estimated to account for 40–50% of all endometrial cancer cases [5, 6]. Additional established risk factors include polycystic ovary syndrome, diabetes mellitus, nulliparity, hypertension and tamoxifen exposure [7]. Among genetic predispositions, pathogenic variants affecting the DNA mismatch repair genes (Lynch syndrome) represent the most significant risk factor for endometrial cancer.

Approximately 40% of endometrial cancers are classified as copy-number low with no specific molecular profile. These tumours are typically low-grade endometrioid carcinomas and are frequently associated with obesity [8]. Other molecular phenotypes identified by The Cancer Genome Atlas Research Network include *Polymerase epsilon (POLE)*-mutant, microsatellite instability (*MMR* deficient) and copy number high p53 abnormal. Together, this molecular framework enables refined risk stratification and directs adjuvant treatment decisions [8, 9].

Endometrial cancer most commonly presents with abnormal uterine bleeding (AUB) including intermenstrual or heavy menstrual bleeding in premenopausal and perimenopausal women and postmenopausal bleeding (PMB) [10, 11]. Approximately 90% of endometrial cancer cases present with PMB; however, only 5-10% of women with PMB are diagnosed with endometrial cancer [12]. The further from menopause that an episode of PMB occurs, the more likely the diagnosis of endometrial cancer [13]. Less commonly, patients may present with non-specific symptoms such as abdominal distention or pain, bladder or bowel dysfunction or haematuria [14, 15].

Surgery with total hysterectomy and bilateral salpingo-oophorectomy remains the mainstay of curative treatment for endometrial cancer, preferably via minimally invasive approaches with lymph node assessment to guide staging and prognosis. Adjuvant therapy, selected according to stage, grade, histology and molecular profile, reduces recurrence in high-risk disease [2, 16, 17]. Conservative management with progestin therapy may be offered to women seeking fertility preservation or to those who are medically unfit for surgery [2, 16]. Disease stage and molecular phenotype are key prognostic markers in endometrial cancer, with advanced-stage and biologically aggressive tumours associated with poorer outcomes. Early detection, particularly of high-risk subtypes, has significant potential to improve survival and to mitigate the impact of the growing burden of the disease on healthcare systems [2, 16, 17].

## Endometrial cancer diagnostic pathway

In the UK, transvaginal ultrasound (TVUS) is the first-line imaging modality for women presenting with AUB [18, 19]. TVUS is minimally invasive, avoids ionising radiation and allows accurate assessment of endometrial thickness (ET) [18, 20]. Women presenting with PMB who have an ET ≤ 4 mm have a less than 1% risk of endometrial malignancy [21, 22]. Provided that cervical malignancy has been ruled out, these women can be reassured and discharged. TVUS is, however, limited by low specificity as a thickened endometrium can also be caused by benign pathologies such as endometrial polyps, fibroids or blood clots in the uterine cavity [20]. Further invasive tests are therefore required to establish a diagnosis in postmenopausal women with an ET > 4 mm [23].

Endometrial sampling, using a suction device such as a Pipelle®, demonstrates good sensitivity and specificity for endometrial cancer detection [24]. However, focal pathologies can be missed as less than 50% of the endometrium is usually sampled [24, 25]. Visualisation of the endometrial cavity with outpatient hysteroscopy allows directed biopsies to be taken when blind endometrial sampling has failed or when focal endometrial pathology is suspected. Hysteroscopic endometrial sampling can fail due to pain, cervical stenosis or inadequate visualisation of the endometrial cavity and can be associated with severe complications including infection and uterine perforation [26, 27].

In symptomatic postmenopausal women, the risk of endometrial cancer in women with an ET ≥ 5 mm is approximately 7% [23, 24]. Despite this relatively low risk, women with a thickened endometrium are required to undergo invasive and painful investigations, with the majority having no or benign pathologies. Distinguishing between those with malignant disease and those without, without relying on invasive endometrial sampling, remains a key research priority for both patients and clinicians. This need has been highlighted by two James Lind Alliance Priority Setting Partnerships, focusing on early cancer detection and endometrial cancer, which ranked the development of accurate, non-invasive or minimally invasive diagnostic tools and improved triage of women with AUB for specialist review, among their top ten priorities [28, 29]. Urine samples are simple to collect and non-invasive. In this review, we present the emerging evidence for the use of urinary biomarkers for the early detection of endometrial cancer.

## Search strategy and selection criteria

A structured literature search was conducted in PubMed (National Library of Medicine interface) and MEDLINE via the Ovid platform (Ovid MEDLINE® ALL) from database inception (i.e., with no date restrictions applied) through June 30, 2025, to identify studies evaluating urinary biomarkers for the diagnosis of endometrial cancer. Search terms combined controlled vocabulary and free-text keywords for “endometrial cancer” or “endometrial carcinoma” with “urine” or “urinary” and biomarker-related terms including “biomarker”, “diagnosis”, “metabolite”, “proteomics”, “protein”, “microRNA”, “extracellular vesicle”, “tumour cell”, “DNA methylation” and “spectroscopy”. Reference lists of relevant articles were also screened.

The search yielded 56 records in MEDLINE and 76 in PubMed. Results were exported as CSV and Excel files, and duplicates were identified and removed electronically. The remaining citations then underwent title and abstract screening. Studies were included if they reported original data on humans evaluating urinary biomarkers for the diagnosis of endometrial cancer. Review articles, non-human studies and studies not involving urine or diagnosis were excluded. Only English-language articles were considered.

Thirty-three studies met the inclusion criteria, comprising seven on metabolites, two on spectroscopy, eight on proteins and peptides, seven on cell-free or extracellular vesicle microRNAs, six on tumour cells and three on DNA methylation. Data extracted included author, study design, biomarker type, marker(s), analytical platform, key findings, urine collection method and country.

## Urine-based biomarkers for endometrial cancer detection

**Fig. 1:**
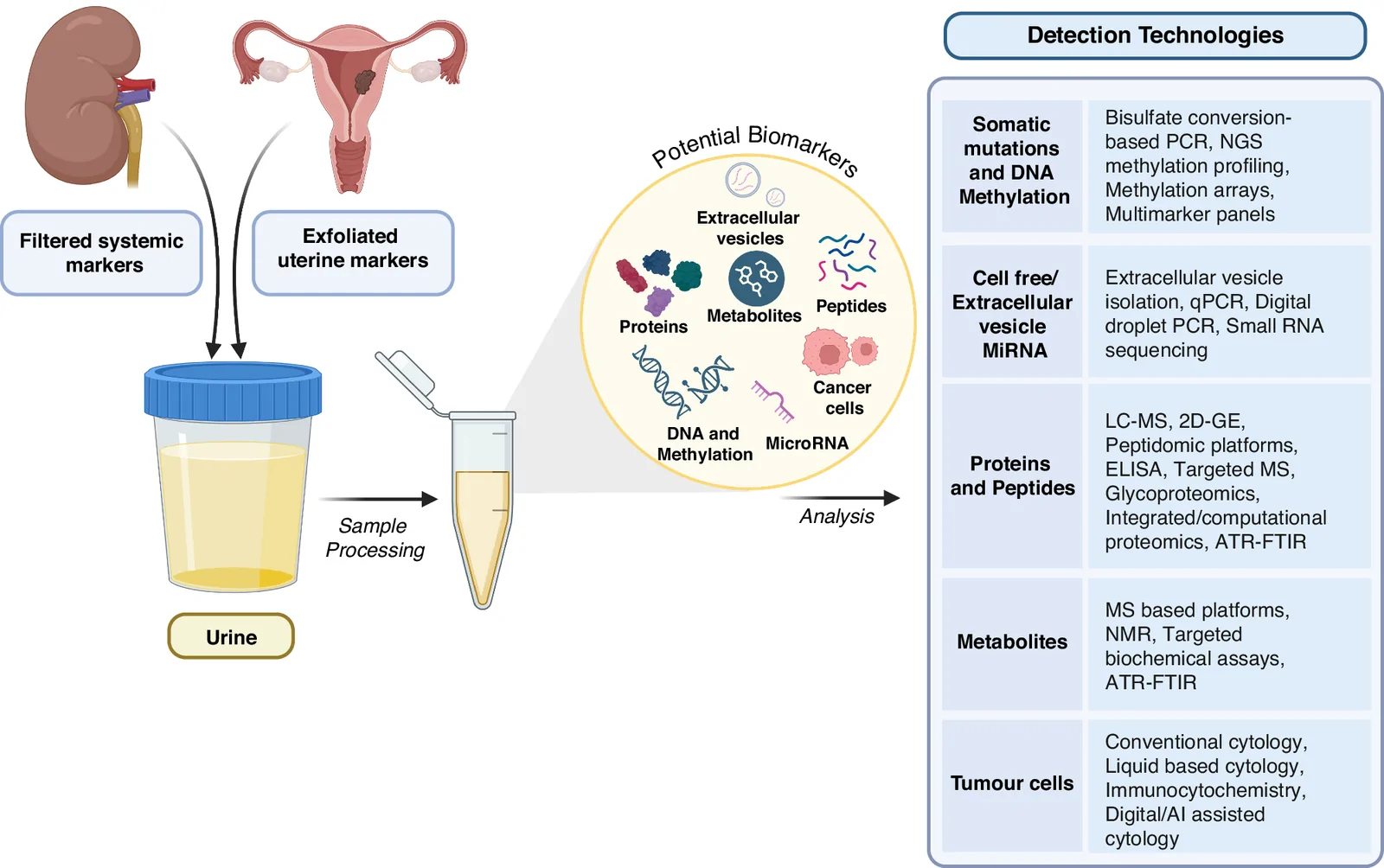
Urinary biomarker sources and detection platforms for endometrial cancer. Urinary biomarkers for endometrial cancer derive from systemic tumour-associated molecules filtered by the kidney and from locally shed uterine tumour material entering the lower genital tract. Urine contains multiple biomarker classes, including metabolites, proteins and peptides, extracellular vesicles, cell-free DNA and microRNAs, DNA methylation markers and exfoliated tumour cells, which can be interrogated using modality-specific analytical platforms for non-invasive cancer detection. Created in https://BioRender.com. Abbreviations: AI: Artificial Intelligence, ATR-FTIR: Attenuated Total Reflectance Fourier-Transform Infrared, DNA: Deoxyribonucleic Acid, ELISA: Enzyme-Linked Immunosorbent Assay, LC-MS: Liquid Chromatography–Mass Spectrometry, MiRNA: MicroRNA, MS: Mass Spectrometry, NGS: Next-Generation Sequencing, NMR: Nuclear Magnetic Resonance, PCR: Polymerase Chain Reaction, qPCR: Quantitative Polymerase Chain Reaction, RNA: Ribonucleic Acid, 2D-GE: Two-Dimensional Gel Electrophoresis.

Urine is an attractive biofluid for endometrial cancer biomarker discovery because it is non-invasive, easy to collect, inexpensive and well suited for repeated sampling [30]. Unlike blood, which is tightly regulated by homoeostatic mechanisms, urine is an excretory biofluid whose composition is less stringently regulated and can therefore more readily reflect physiological and pathological changes. Furthermore, the urinary proteome has lower complexity and a narrower dynamic range than that of blood, facilitating the detection of disease-associated and low-abundance biomarkers [31, 32]. Although systemic biomarkers may be present at low abundance in early disease, urine provides a unique opportunity for biomarker discovery by capturing molecules derived from systemic circulation through renal filtration and locally derived biomolecules from the urogenital tract (Fig. 1). Many urinary metabolites and low–molecular weight proteins and peptides are derived from circulating molecules that undergo renal filtration and tubular processing allowing urine to reflect systemic metabolic and proteomic alterations [33–35]. Nucleic acids such as cell-free DNA, microRNAs (miRNAs) and extracellular vesicles (EVs) can derive from both systemic circulation and direct shedding of tumour material into the genital tract [36–38]. Exfoliated malignant cells almost exclusively originate from local shedding of endometrial tissue through the cervix into the vagina, contaminating the urine and are most commonly observed in symptomatic women [39]. This is particularly valuable in endometrial cancer, where around 90% of patients present with AUB [12], creating a clinical window to capture tumour-derived material in urine.

The dual origin of urinary biomarkers for endometrial cancer detection has important implications for biomarker discovery, validation, and clinical translation. While cervicovaginal material can contribute genuine biological signals, it may also introduce confounding variability that affects biomarker specificity and reproducibility [36–38]. Distinguishing between systemically derived and locally shed biomarkers, while also accounting for potential confounding from cervicovaginal secretions, is therefore an important consideration when interpreting urinary signals. Although not required in all settings, complementary sampling strategies, such as comparison with cervicovaginal fluid or matched biofluids, may help clarify biomarker origin where overlapping signals could confound interpretation.

A recent study from our group addressed this using a two-pronged biomarker discovery approach, in which SWATH-MS urinary proteomic datasets were searched against both a human plasma spectral library and a bespoke endometrial cancer cervicovaginal fluid spectral library. This enabled distinction between biomarkers likely to be renally excreted and those arising from local shedding from the uterus and lower genital tract, demonstrating that urinary biomarkers may reflect overlapping biological sources and should not be interpreted independently of their origin [34].

Modern analytical platforms now enable comprehensive urine-based biomarker analysis across multiple domains: cytology and single-cell imaging, genomics and epigenomics (polymerase chain reaction (PCR), NGS, methylation assays), proteomics (liquid chromatography-mass spectrometry (LC-MS), immunoassays), metabolomics (LC-MS, GC-MS, nuclear magnetic resonance (NMR)) and EV analytics (Fig. 1) [40]. These complementary approaches provide multi-dimensional insights into tumour biology and have the potential to improve diagnostic sensitivity when integrated compared to single modality analyses. However, successful clinical translation depends on rigorous attention to pre-analytical variables, including collection timing, urine fractionation, preservatives, handling, storage and the transparent reporting of these factors. Variability and incomplete reporting at these stages can undermine reproducibility, bias estimates of diagnostic accuracy and hinder clinical adoption [30].

Before a candidate biomarker can be adopted in clinical practice, it must progress through sequential phases, from preclinical exploratory studies to assay development, retrospective and prospective validation and ultimately cancer control evaluation [41]. Key performance measures include analytical validity (accuracy and reproducibility), clinical validity (ability to correctly classify disease, typically expressed as sensitivity, specificity, PPV and NPV) and clinical utility (impact on outcomes, safety, acceptability and cost-effectiveness) [42]. For screening biomarkers, high sensitivity is prioritised to detect early disease and minimise false negatives, whereas specificity becomes critical during diagnostic confirmation to avoid unnecessary interventions and reduce patient burden [43, 44].

## Somatic mutations and DNA methylation markers

**Fig. 2:**
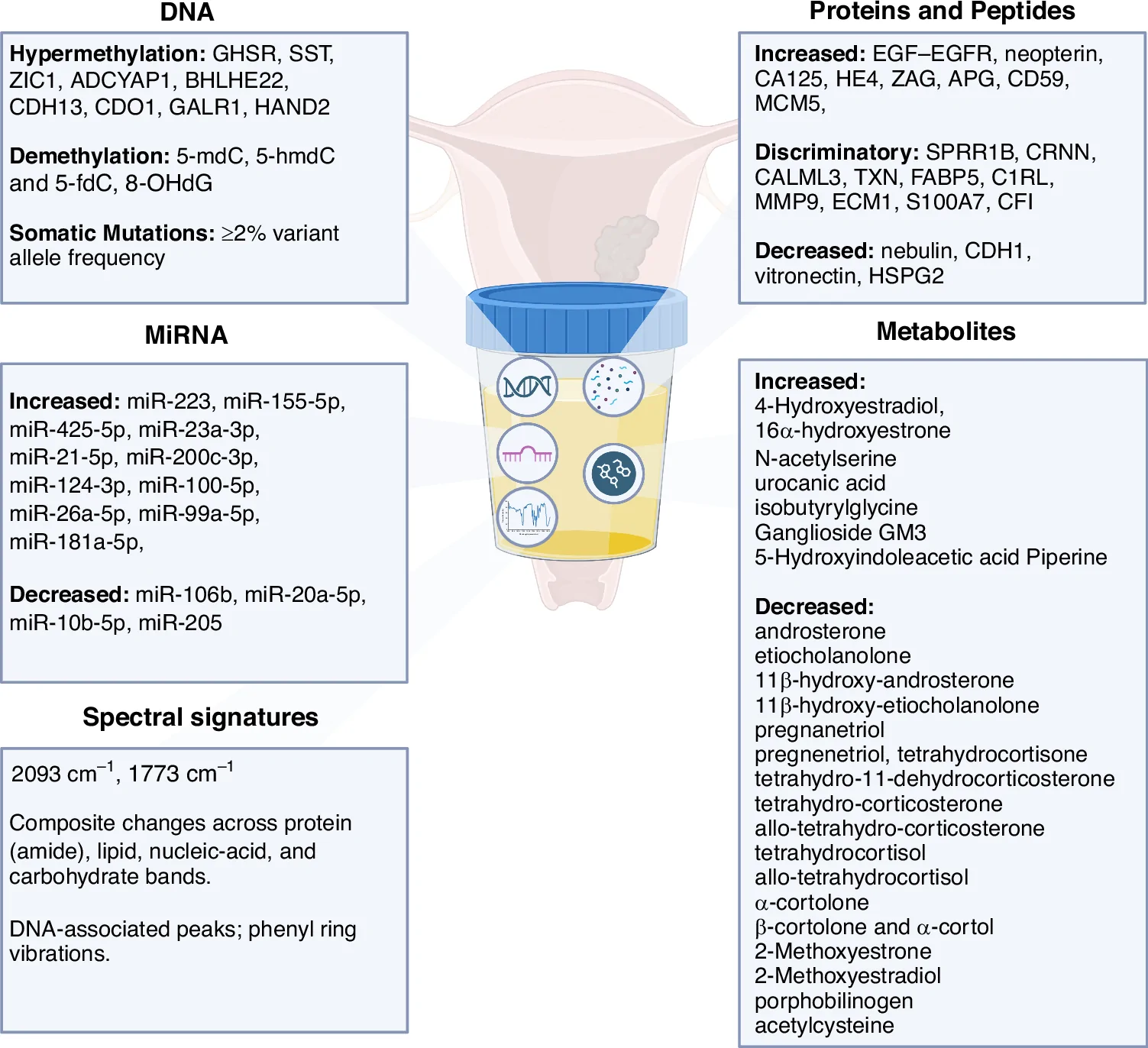
Urine biomarkers for endometrial cancer detection. Summary of differentially abundant metabolites, microRNAs (miRNAs), proteins/peptides and DNA features identified in urine samples in this review. Created in https://BioRender.com. Abbreviations: 5-fdC: 5-formyl-2′-deoxycytidine, 5-hmdC: 5-hydroxymethyl-2′-deoxycytidine, 5-mdC: 5-methyl-2′-deoxycytidine, 8-OHdG: 8-hydroxy-2′-deoxyguanosine, A1AG/AGP: alpha-1-acid glycoprotein, ADCYAP1: adenylate cyclase-activating polypeptide 1, BHLHE22: basic helix-loop-helix family member E22, C1RL: complement C1r-like protein, CA125: cancer antigen 125 (MUC16), CALML3: calmodulin-like protein 3, CD59: CD59 glycoprotein, CDH1: E-cadherin, CDH13: cadherin-13, CDO1: cysteine dioxygenase type 1, CFI: complement factor I, CRNN: cornulin, ECM1: extracellular matrix protein 1, EGF: epidermal growth factor, EGFR: epidermal growth factor receptor, FABP5: fatty acid-binding protein 5, GALR1: galanin receptor 1, GHSR: growth hormone secretagogue receptor, HAND2: heart- and neural-crest derivatives-expressed protein 2, HE4: human epididymis protein 4, HSPG2: heparan sulfate proteoglycan 2 (perlecan), MCM5: minichromosome maintenance protein 5, miR: microRNA, MMP9: matrix metalloproteinase-9, Nebulin: nebulin, S100A7: S100 calcium-binding protein A7, SPRR1B: small proline-rich protein 1B, SST: somatostatin, TXN: thioredoxin, VTN: vitronectin, ZAG: zinc-α2-glycoprotein, ZIC1: Zic family member 1.

Somatic mutations and DNA methylation in urine have emerged as promising non-invasive biomarkers for endometrial cancer detection (Fig. 2). Using targeted NGS of a 47 gene panel, Costas et al. detected tumour specific mutations in 100% of urine supernatant samples from 19 endometrial cancer cases, with ≥2% variant allele frequency present in only 5% of 20 controls. Urine supernatant showed the highest mutation burden with strong concordance with matched tumour tissue (94%), enabling accurate molecular subtyping [45]. This demonstrates that urine has the potential to capture tumour-derived DNA from the uterine cavity, providing a complementary signal to methylation markers for detection and risk stratification.

Epigenetic alterations, particularly promoter hypermethylation of tumour suppressor genes, occur early in endometrial carcinogenesis and can serve as sensitive biomarkers [46]. A feasibility study identified hypermethylation of GHSR, SST and ZIC1 in urine from 42 cases and 46 controls, with full void urine achieving an AUC of 0.95 [47]. A subsequent larger study expanded the panel to nine genes (ADCYAP1, BHLHE22, CDH13, CDO1, GALR1, GHSR, HAND2, SST, ZIC1), consistently hypermethylated across urine, cervicovaginal and cervical samples, with seven markers performing best in urine [48]. Multi-gene panels outperformed single markers, reflecting the molecular heterogeneity of endometrial cancer and improving diagnostic robustness [48]. Additional genomic/epigenomic urinary markers include altered DNA demethylation products, specifically 5-methyl-2′-deoxycytidine (5-mdC), 5-hydroxymethyl-2′-deoxycytidine (5-hmdC) and 5-formyl-2′-deoxycytidine (5-fdC), which are intermediates in the active DNA demethylation pathway, alongside the oxidative DNA damage marker 8-hydroxy-2′-deoxyguanosine (8-OHdG). These markers show moderate diagnostic performance (AUC 0.667-0.844) and may serve as adjuncts to methylation panels rather than standalone biomarkers [36].

The Women’s cancer risk identification–quantitative polymerase chain reaction test for endometrial cancer (WID-qEC), a DNA methylation–based assay that analyses targeted methylation regions within the GYPC and ZSCAN12 genes in cervicovaginal samples, has demonstrated strong real-world performance in the UK, with sensitivities exceeding 90% in women presenting with AUB [49]. This result highlights the potential of methylation markers to enable the non-invasive detection of endometrial cancer, and it will be important to explore how the test performs in urine, where similar epigenetic signals may be detectable in a different biochemical context.

Despite promising findings of urine methylation studies, limitations remain. Most studies used case-control or enriched cohorts, potentially overestimating performance. DNA methylation can be influenced by age, obesity, inflammation and hormonal status and pre-analytical factors such as urine fraction, volume and DNA yield were not routinely standardised [50]. Large prospective studies with external validation are required to establish clinical utility of these markers and to facilitate routine implementation.

## Urinary miRNAs and extracellular vesicles in endometrial cancer

MiRNAs are small non-coding RNAs that regulate gene expression post-transcriptionally and are frequently dysregulated in endometrial cancer, where they influence proliferation, invasion, epithelial-to-mesenchymal transition (EMT) and immune pathways [51]. Extracellular vesicles (EVs) are small membrane-bound particles released by cells that carry proteins, RNA, miRNAs and other molecules to enable communication between cells [52]. Urinary EVs were first detected by Pisitkun et al. [53] and can be detected in urine from systemic glomerular filtration [54] or through local shedding [39]. Subsequent studies have shown that urinary miRNAs, including those encapsulated within EVs, are detectable and stable, supporting their potential utility as liquid biopsy targets.

While urinary miRNAs have been extensively studied in prostate and bladder cancers [55], evidence in endometrial cancer remains limited. Small exploratory cohorts report downregulation of miR-106b and upregulation of miR-223 in urine, distinguishing cases from controls independent of age, BMI or comorbidities [56, 57] (Fig. 2). Further validation in larger cohorts is essential for translation.

Several studies have investigated EV-derived miRNAs in endometrial cancer, highlighting their potential as non-invasive biomarkers. Ruskin et al. analysed exosomal miRNAs in a small cohort, identifying ten miRNAs upregulated (up to 30-fold) and two downregulated (2-fold), with miR-155-5p most overexpressed and miR-20a-5p most under expressed [58]. Srivastava et al. further identified miR-200c-3p as a potential diagnostic biomarker in endometrial cancer [37] (Fig. 2). Biologically, miR-155 is a well-characterised oncomiR implicated in inflammatory signalling, immune modulation, mismatch repair expression and tumour progression [59, 60], whereas miR-20a, part of the miR-17-92 cluster, regulates the cell cycle and exhibits tumour-suppressive activity in endometrial cancer [61, 62]. The miR-200 family also plays a central role in epithelial-to-mesenchymal transition (EMT) and tumour differentiation [63, 64]. These functional relationships lend strong biological plausibility to their enrichment in urinary EVs.

Conversely, Zavesky et al. reported no statistically significant differences in exosomal miRNAs between cases and controls [65], underscoring the variability observed across studies. More recently, Ritter et al. used miRNA microarrays to profile serum and urine exosomal miRNAs across breast, ovarian and endometrial cancers. In endometrial cancer, miR-484 and miR-23a were downregulated in serum, whereas miR-205 was selectively downregulated in urine [66] (Fig. 2). Given the established role of miR-205s in EMT regulation and endometrial epithelial integrity [63, 64], its urine-specific dysregulation supports the presence of a compartmentalised, tumour-derived signal. The lack of concordance between serum and urine miRNAs emphasises the need to validate urinary biomarkers independently rather than extrapolating from blood-based studies.

This discrepancy likely reflects fundamental biological differences between biofluids, as circulating miRNAs in serum predominantly capture systemic tumour and host responses, whereas urinary miRNAs arise from renal filtration and contributions from cells within the urinary and genitourinary tract [67]. The passage of miRNAs into urine is not a passive process. Filtration at the glomerulus, together with tubular transport and secretion, can influence which miRNA species are retained or excluded compared with blood [68]. In addition, miRNA stability and detectability are influenced by their mode of packaging, with EVs and protein complexes protecting miRNAs from degradation. This is particularly important in urine, where relatively high RNase activity may preferentially preserve and enable detection of protected miRNA species, such as those encapsulated within extracellular vesicles or associated with RNA-binding proteins, which may differ from circulating serum miRNA profiles [69, 70]. These differences suggest that urinary miRNA profiles are biologically distinct from their circulating counterparts and should be interpreted within their own context.

Systematic reviews further highlight consistent miRNA dysregulation in endometrial cancer, including upregulation of oncogenic miRNAs (miR-182, miR-183, miR-200 family, miR-205, miR-223) and downregulation of tumour-suppressive families such as miR-34 and miR-152 [57, 63]. Although most evidence originates from tissue and plasma, the central role of these miRNAs in malignant processes makes them compelling candidates for urinary biomarker development, pending direct urine-based validation.

Methodological rigour remains a critical determinant of progress in urinary miRNA research. EVs protect miRNA cargo and may enhance tumour specificity, yet isolation methods are highly variable, frequently co-isolating proteins and other contaminants, thus complicating quantification [71]. Standardised protocols for EV capture, characterisation and data normalisation are lacking [72–75] and current technologies do not support high-throughput, clinically scalable EV isolation, limiting translational potential. Cell-free urine miRNAs are similarly influenced by haematuria, inflammation, renal function and patient heterogeneity, contributing to inconsistent results and poor concordance with tissue or plasma profiles [75]. While cell-free studies typically identify single candidates and EV-based approaches yield broader signatures [67], neither approach has yet produced reproducible, clinically actionable biomarkers. Future progress will depend not only on standardised pre-analytical workflows, transparent reporting, multicentre validation and data-driven panel refinement, but also on recognising urinary miRNAs as a distinct compartment. Biomarker development should therefore focus on urine itself, with clear differentiation of miRNA fractions (e.g., EV-associated versus cell-free), rather than relying on signatures derived from blood, to support the development of clinically applicable urine assays.

## Spectroscopy-based urinary biomarkers

Spectroscopy offers a comprehensive assessment of urinary biochemical composition, complementing metabolomics and proteomics, which detect specific disruptions in amino acid, lipid, nucleotide and protein pathways. Attenuated total reflection Fourier-transform infrared (ATR-FTIR) spectroscopy captures these alterations as a single spectral “fingerprint,” providing a rapid, low-cost, multi-analyte approach alongside targeted assays [76].

Early studies by Paraskevaidi et al. demonstrated that mid-infrared urinary spectra could differentiate endometrial cancer, ovarian cancer and healthy controls, driven by reproducible changes in nucleic acid, lipid and protein-associated spectral regions. Using supervised machine learning, diagnostic sensitivity and specificity exceeded 90% for endometrial cancer detection [77]. These findings were validated by Meza Ramirez et al. in a larger cohort of 109 endometrial cancer cases and 110 benign controls, reporting sensitivities and specificities above 95% and overall diagnostic accuracy of 99.3% [78]. Remarkably, discrimination of endometrioid endometrial cancer from controls reached 100% for all parameters. Additionally, urinary spectral signatures could differentiate endometrioid from non-endometrioid tumours, suggesting some capacity for molecular stratification and could also differentiate Stage IA grade 1 endometrial cancer from controls. Notably, spectral biomarkers consistent with DNA, phospholipids and phenyl ring vibrations were enriched in the cancer group, reflecting abnormalities in genomic stability, membrane biosynthesis and protein conformation [78].

Most recently, Vigo et al. applied advanced machine learning classifiers to ATR-FTIR urine spectroscopy, enabling discrimination of multiple gynaecological cancers and breast cancer from healthy controls [79]. Highly discriminatory wavelengths were identified (2093 cm⁻¹ and 1773 cm⁻¹) associated with protein backbone and lipid ester vibrations (Fig. 2). Their models achieved >91% accuracy for differentiating gynaecological cancer from controls, although discrimination for endometrial cancer specifically was not found [79]. These results highlight both the promise and limitations of spectroscopy-based approaches: while highly sensitive to malignant biochemical signatures, spectral overlap among gynaecological cancers may limit tumour specific classification.

Overall, urinary ATR-FTIR provides reproducible, discriminant signatures that reflect composite biochemical alterations in endometrial cancer. The short analysis time, limited sample handling and potential for automated processing suggest that ATR-FTIR could be incorporated into high throughput triage pathways. Standardised spectral acquisition protocols, along with prospective evaluation in diverse populations, remain necessary before any clinical application can be established [76].

## Urinary proteins and peptides in endometrial cancer

Urinary proteomics and peptidomics have moved from proof-of-concept single markers to biologically integrated, multiplex signatures with stronger diagnostic performance (Table 1). Early single-protein studies established plausibility in this space.

**Table 1:** Urine-based (and related) biomarkers for endometrial cancer: evidence summary table.

Author, year	Study Design	Validation	Type of Marker	Marker	Analytical method	Findings	Sample Collection	Country
Helder et al. [126] 2020	Case control EC:42 Control: 46	None reported	DNA Methylation (urine only)	Genes GHSR, SST and ZIC1	Methylation PCR.	Increased methylation level of GHSR, SST and ZIC1 in EC compared to controls, with highest discriminatory power in full void urine samples. AUC for EC detection: 0.86-0.95	Full void urine, urine sediment and urine supernatant were tested and compared	Netherlands
Wever et al. [48] 2023	Case control EC: 103 Control: 317	Yes (internal only) Using leave-one out cross validation	DNA Methylation (urine, cervicovaginal self-samples and cervical scrapes)	Promoter hypermethylation of the ADCYAP1, BHLHE22, CDH13, CDO1, GALR1, GHSR, HAND2, SST and ZIC1 genes	Quantitative methylation-specifc PCR (qMSP)	Methylation levels of ADCYAP1, BHLHE22, CDH13, CDO1, GALR1, GHSR, HAND2, SST and ZIC1 were significantly higher in urine, cervicovaginal and cervical scrapes for EC patients. Combination of marker panels gives more accurate result.	Full void urine collected in collection tubes containing 0.6 M ethylenediaminetetraacetic acid at home	Netherlands
Zhao et al.[36] 2024	Case control EC: 37 Control: 39	Yes (internal /methodological only)	Nucleic acid modification	5 mU, 5-mdC, 5-hmdC, 5-fdC and 8-OHdG	Dispersive solid-phase extraction (DSPE) + UHPLC-QE-Orbitrap-MS/MS and HPLC-UV	Levels of 5-mdC, 5-fdC and 8-OHdG in urine from EC patients were elevated, while 5-hmdC was significantly reduced. AUC 0.667-0.844	Morning urine sample	China
Costas et al. [45] 2023	Case control: *Targeted approach* EC: 19 Control: 20	Partial (paired tissue comparison)	DNA (urine only)	Somatic mutations (VAF ≥ 2%)	NGS with molecular barcodes	DNA mutations were detected in 100% of EC cases. Only 1 control (5%) had detectable variants	At outpatient clinic, first-fraction urine collection device Colli-Pee® (DNA Genotec) used to collect urine.	Spain
Zavesky et al. [56] 2015	Case control *Targeted approach* EC: 10 Other cancers and healthy controls	None reported	Cell free micro-RNA Extracellular vesicle (EV) RNA (urine only)	Eighteen different micro-RNAs	qRT-PCR	Cell free miR106b was down regulated in EC cases No difference was found when tested for exosome RNA.	Second morning void	Czechia
Ruskin et al. [58] 2016	Case control: EC: 12 Control: 5	None reported	Extracellular vesicle (EV) RNA (urine only)	Twelve different micro-RNAs	qPCR	Ten micro-RNAs had at least 30-fold increased expression in EC and 2 were downregulated in EC cases.	Single time-point urine sample (pre-surgical)	USA
Srivastava et al. [37] 2018	Case control: *Targeted approach* EC: 22 Control: 5	None reported	Extracellular vesicle (EV) RNA (urine only)	84 most abundantly expressed and biologically significant miRNAs curated in miRbase		Elevated Has-miR-200c-3p in EC cases.	30-50 mL urine in sterile pot prior to surgery	USA
Ritter et al. [66] 2020	Case control: *Untargeted approach* EC: 10 Ovarian cancer: 10 Breast cancer: 25 Control: 30	Internal only (limited) Independent RT-qPCR set.	miRNA (urine and serum)	Expression levels of 2549 human miRNAs specified by the latest version of miRBase database.	miRNA microarray and RT-qPCR	miR-10b-5p was upregulated in EC cases, miR-205-5p was down-regulated in EC cases.	Single time-point urine and blood sample (pre-surgical)	Germany
Donkers et al. [57] 2021	Case control *Targeted approach* EC: 24 Control: 37	None reported	miRNA (urine only)	A total of 41 different microRNAs were analysed	qRT-PCR	Higher expression levels of miR-223 in EC cases (*p* = 0.003).	Single time-point 100 mL urine in sterile pot prior to surgery	UK
Paraskevaidi et al. [77] 2018	Case control *Untargeted approach* EC: 10 Ovarian cancer: 10 Control: 10	Internal only Multivariate models (classification algorithms). No specific validation cohort	Spectroscopy (urine only)	Spectral biochemical signatures (IR absorbance features)	Attenuated Total Reflectance Fourier-Transform Infrared (ATR-FTIR) spectroscopy	Urinary infrared spectral signatures discriminated both cancers from controls. EC: 95% sensitivity, 100% specificity, 95% accuracy. Ovarian cancer: 100% sensitivity, 96.3% specificity, 100% accuracy.	Pre-op urine from catheter after fasting for >6 hours	UK
Meza Ramirez et al. [78] 2022	Case control *Untargeted approach* EC: 109 Control (benign gynaecological conditions): 110	Internal only Statistical/chemometric classification methods No specific validation cohort	Spectroscopy (urine only)	Spectral biochemical signatures (IR absorbance features)	ATR-FTIR spectroscopy and chemometric/statistical modelling	Identified spectral biomarkers (DNA bands, phenyl vibrations) strongly predicted EC: 95.29% sensitivity, 98.89% specificity, accuracy: 99.32%) EC (endometrioid) vs controls: Sensitivity 100%, Specificity 100% AUC: 100% Endometrioid vs non-endometrioid EC: Sensitivity 100%, Specificity 100% AUC: 100% Stage IA grade 1 EC vs controls: Sensitivity 100%, Specificity 100% AUC: 100% Stage I EC vs controls: Sensitivity 98.11%, Specificity 97.36% AUC of 99.73%	urine collected via catheter post anaesthesia (fasted >6 h)	UK
Vigo et al. [79] 2025	Case control *Untargeted approach* EC: 32 Other cancers (breast, cervical, ovarian, vulvar): 71 Control: 206	Internal only 10-fold cross-validation after LASSO feature selection and model training	Spectroscopy (urine only)	IR spectral features (not molecules)	(ATR-FTIR) spectroscopy	Accuracy > 91% for classifying cancer vs controls. Identified discriminant wavelengths: 2093 cm⁻¹ and 1773 cm⁻¹. Not EC specific.	Single time-point urine sample	Switzerland
Cole et al. [90] 1988	Case control: *Targeted approach* gynaecological cancer: 87 (EC not specified) Control: 50	None reported	Peptide (urine and serum)	Beta-core fragment of hCG	Immunoradiometric assay	77% of endometrial cancer cases had detectable urinary UGF ( > 0.2 ng/mL).	Single time-point urine and serum.	USA
Kinugasa et al. [91] 1995	Case control: *Targeted approach* EC: 37 Other gynaecological cancer: 242 Benign disease: 207	None reported	Peptide (urine and serum)	Beta-core fragment of hCG	Immunoassay for intact hCG, free β-hCG, β-CF	Upregulated in 37.8% of EC patients. Urine β-CF detection higher than serum hCG in all cancers.	Single time-point urine and serum.	Japan
Mattila et al. [80] 1988	Cross-sectional Case control Total of 97 patients. Unspecified number of EC cases, controls and various other cancers	None reported	Proteins (urine only)	Epidermal Growth Factor (EGF), immunoreactive form (U-irEGF)	Radioimmunoassay	Elevated U-irEGF in EC compared to controls (*p* = 0.0005)	Single time-point urine sample (pre-surgical)	Finland
Bazzett et al. [83] 2003	Case control *Targeted approach* EC: 31 Control: 19 Other gynaecological cancers: 65	None reported	Proteins (urine only)	matrix metalloproteinases (MMPs) MMP-2 and MMP-9	Substrate gel electrophoresis (zymography)	No associations with MMPs and EC.	Single time-point urine sample (pre-surgical)	USA
Mu et al. [87] 2012	Case control *Untargeted approach* EC: 7 (stages IB and IIA) Control: 11	Technical only CGB lectin affinity chromatography + LC–MS/MS analysis No specific validation cohort	Proteins (urine only)	zinc alpha-2 glycoprotein, alpha 1-acid glycoprotein, CD59 and nebulin	Discovery via 2-DE + lectin blot Validation only for one biomarker (nebulin fragment)	Elevated zinc alpha-2 glycoprotein, alpha 1-acid glycoprotein in EC cases. Reduced CD59 and Nebulin in EC patients.	Single time-point urine sample (pre-surgical, morning)	Malaysia
Mu et al. [33] 2016	Case control *Targeted approach* EC: 4 Control: 4 Ovarian cancer: 4 Cervical cancer: 4 (All stages IB and IIA)	None reported	Peptides (urine only)	Glycopeptide m/z 1449	CMB (mannose-binding) lectin chip Surface-Enhanced Laser Desorption/Ionisation Time-of-Flight MS (SELDI-TOF MS)	Glycopeptide m/z 1449 can differentiate EC from ovarian, cervical cancer and healthy controls.	Single time-point urine sample (pre-surgical, morning)	Malaysia
Bostanci et al. [84] 2017	Case control *Targeted approach* EC: 41 Control: 41	None reported	Proteins (serum and urine)	Neopterin	Urinary neopterin: HPLC Serum neopterin: ELISA	Urinary neopterin elevated in EC vs controls (p < 0.001). Higher urinary neopterin in advanced EC (III–IV) vs early EC (I–II) (p < 0.01). Serum neopterin not discriminatory.	Single time-point urine sample (first void, pre-surgical) Single time-point serum sample (morning, pre-surgical)	Turkey
Kacírová et al. [127] 2019	Case control *Untargeted approach* EC: 5 (Type I and II) Control: 7	None reported	Proteins (urine only)	Multiple proteins were assessed, with particular interest in basement membrane-specific heparan sulfate proteoglycan core protein (HSPG2), vitronectin (VTNC) and Cadherin-1 (CDH1)	nano-scale-HPLC electrospray ionisation, tandem mass spectrometry (nano-HPLC-ESI-MS/MS)	Down-regulation of VTNC and CDH1 in EC cases compared to controls.	Two morning urine samples (pre-surgical)	Czechia
Stockley et al. [89] 2020	Case control *Targeted approach* EC: 41 Ovarian cancer: 23 Endometrioid, serous, clear cell, carcinosarcoma, Adenocarcinoma NOS, all grades/stages) Control: 58 (benign gynaecological conditions)	No	Protein (urine sediment and vaginal)	MCM5	MCM5-ELISA	Elevated MCM5 level in EC (AUC 0.83, Sensitivity: 87.8%, Specificity: 75.9%) and ovarian cancer (0.68) compared to controls.	Full void urine. Vaginal swab collected by the research nurse or a vaginal tampon worn 6–8 h.	UK
Njoku et al. [34] 2023	Case control EC: 50 Endometrioid, non-endometroid, all grades/stages) Control: 54	Internal only model-development level No specific validation cohort	Proteins (urine only)	A total of 798 renally excreted endometrial cancer-related systemic biomarkers.	SWATH-MS + machine learning	Combination of the 10-marker panel (SPRR1B, CRNN, CALML3, TXN, FABP5, C1RL, MMP9, ECM1, S100A7 and CF1) showed better accuracy, with AUC 0.92, sensitivity of 83.7% and specificity of 83.9%	Self-collected urine in sterile pot	UK
Aitokallio-Tallberg et al. [99] 1987	Case control *Targeted approach* EC: 12 Control: 12 Other gynaecological tumour cases: 23 Benign gynaecology cases: 23	None reported	Metabolites (urine only)	6-keto-prostaglandin F1a	High performance liquid chromatography (HPLC) Followed by radioimmunoassay (RIA) for quantification	6-keto-prostaglandin F1a levels do not differ between EC and control groups	pre-operatively collected urine.	Finland
Bufa et al. [100] 2010	Case control *Targeted approach* EC: 13 Control: 10	None reported	Metabolites (urine only)	23 different steroid metabolites. (androgens, progesterone, corticoids)	Gas chromatography–mass spectrometry (GC–MS) with selected ion monitoring	Reduced levels of 15 different steroid metabolites, including androsterone, etiocholanolon, 11β-hydroxy-androsterone, 11β-hydroxy-etiocholanolon, pregnanetriol in EC cases.	Urine collected over 24 h.	Hungary
Zhao et al. [101] 2015	Case Control *Targeted approach* EC: 23 Control: 23	None reported	Metabolites (urine only)	Oestrogen metabolites 2-hydroxyestradiol (2-OHE2), 2-hydroxyestrone (2-OHE1), 4-hydroxyestradiol (4-OHE2), 4-hydroxyestrone (4-OHE1), 16α-hydroxyestrone (16α-OHE1), 2-methoxyestradiol (2-MeOE2) and 2-methoxyestrone (2-MeOE1)	LC-MS with hollow-fibre liquid-phase microextraction (HF-LPME) for enrichment.	Elevated 4-OHE2 in EC cases Reduced 2-MeOE1 and 2-MeOE2 in EC cases	Urine collected over 24 h using 1 L bottle with 1 g ascorbic acid	China
Shao et al. [106] 2016	Case control *Untargeted approach* EC: 25 Control: 25 Hyperplasia: 10	Internal only Verification with support vector machine (SVM) predictive modelling	Metabolites (urine only)	Porphobilinogen, acetylcysteine, N-acetylserine, Urocanic acid, Isobutyrylglycine.	Ultra-Performance LC-MS Quadrupole Time-of-Flight MS (UPLC-Q-TOF/MS)	5-metabolite biomarker panel distinguishing EC from controls. Reduced porphobilinogen and acetylcysteine, elevated N-acetylserine, Urocanic acid, Isobutyrylglycine	pre-operatively collected urine (morning)	China
Yi et al. [110] 2022	Case control *Untargeted approach* EC: 44 (grade 1 or 2 endometrioid EC) Control: 43 (normal endometrium but suffers from other gynaecological disease, unsure about bleeding status)	No	Metabolites (urine, tissue, intrauterine brushings)	Multiple metabolites and 13 significant metabolic pathways	Untargeted LC-MS metabolomics and proteomics.	Most metabolites were downregulated in urine samples but upregulated in endometrial and intrauterine brushing samples. AUC for urine with eh top 100 metabolites is 0.808.	pre-operatively collected urine (morning, fasted) Endometrial tissue was collected after surgical intervention. Intrauterine brushings collection timing unspecified.	China
Chen et al. [108] 2024	Case control *Untargeted approach* EC: 146 (79 low-risk, 67 high-risk) (124 type I and 22 type II) Control: 59 (abnormal uterine bleeding)	Internal only Internal validation with ROC in discovery and validation cohorts.	Metabolites (urine and serum)	Amino acid, nicotinamide, purine, glutamine, folic acid	Ultra-performance LC-MS (UPLC-MS).	Metabolites involved in synthesis and degradation of valine, leucine and isoleucine were elevated in EC cases. Purine metabolism was higher in high-risk than low-risk EC and glutamine levels were lower in high-risk EC. Folic acid levels were lower in Type II compared with Type I EC. Most accurate diagnostic performance e in combined urine and serum. AUC values: Urine 0.902, Serum 0.821, Combined 0.953 (discovery) and 0.976 (validation).	pre-operatively collected urine and serum (morning, fasted)	China
Fu et al. [35] 2024	Case control *Untargeted approach* Early EC: 110 Control: 110	None reported	Metabolites (urine and serum) and hub genes	Untargeted multi-omics approach. 83 differential metabolites: 10 prioritised as potential biomarkers. 19 hub genes identified: 3 prioritised as top candidates.	LC‑MS metabolomics + transcriptomics (GEO datasets).	3-metabolite combination (histamine, 1-methylhistamine and methylimidazole acetaldehyde) as well as combination of RRM2, TYMS and TK1 discriminated EC from controls.	50 patients and 50 controls provided first-morning urine sample. 60 patients and 60 controls provided blood sample (fasted)	China
Kanno et al. [116] 2002	Single case report Postmenopausal woman with endometrial (corpus) cancer. Presented with positive urine cytology	n/a	Cytology	Exfoliated EC cells	Cytology → Unspecified	Positive urine cytology	Single void urine cytology.	Japan
Khan et al. [117] 2011	Single case report Localised uterine malignancy presenting with positive urine cytology	n/a	Cytology	Exfoliated EC cells	Cytology → Unspecified	Positive urine cytology in microscopic haematuria	Single void urine cytology.	UK
Wang et al. [118] 2015	Single case report Atypical squamous cells (ASC) in urine leading to EC diagnosis	n/a	Cytology	Exfoliated EC cells	Cytology → Papanicolaou stain	Atypical squamous cell found in symptomatic patient with haematuria	Single void urine cytology.	USA
Betancourt et al. [119] 2021	Single case report EC first identified through voided urine cytology	n/a	Cytology	Exfoliated EC cells	Cytology → cell block → IHC.	Cells showed papillary morphology with marked pleomorphism, high nuclear-to-cytoplasmic ratios, nuclear hyperchromasia, coarse chromatin and occasional cytoplasmic vacuoles. IHC positive for PAX8 with focal, weak staining for oestrogen receptor, negative for GATA3	Unspecified	USA
O’Flynn et al. [39] 2021	Case control EC: 103 Control: 113	Yes Internal (within-study diagnostic accuracy) and partial external (performance assessed in two clinically distinct populations: known cancer cases and a real-world symptomatic PMB cohort)	Cytology (urine and vaginal)	Exfoliated EC cells	Cytology → Papanicolaou staining	91.7% sensitivity and 88.8% specificity for combined urine and vaginal cytology. Vaginal cytology was more sensitive (90.2% versus 72.0%), but less specific (88.7% versus 94.9%) than urine cytology for EC detection	Voided urine + Delphi-screener vaginal samples.	UK
Narine et al. [120] 2022	Single case report Presenting with dual malignancies (uterine carcinosarcoma + bladder urothelial carcinoma)	n/a	Cytology	Exfoliated cancer cells (urine and vaginal)	Cytology → IHC.	Urine contained more urothelial malignant cells and less adenocarcinoma cell clusters, vaginal samples showed the reverse patterns	Self-collected urine in a sterile pot	UK

*2-DE* Two-Dimensional Gel Electrophoresis, *5-fdC* 5-formyl-2′-deoxycytidine, *5-hmdC* 5-hydroxymethyl-2′-deoxycytidine, *5-mdC* 5-methyl-2′-deoxycytidine, *5-mU* 5-methyluridine, *8-OHdG* 8-hydroxy-2′-deoxyguanosine, *ASC* Atypical Squamous Cells, *ATR-FTIR* Attenuated Total Reflectance Fourier-Transform Infrared, *AUC* Area Under the Curve, *DSPE* Dispersive Solid-Phase Extraction, *EC* Endometrial Cancer, *ELISA* Enzyme-Linked Immunosorbent Assay, *EV* Extracellular Vesicle, *GC* Gas Chromatography, *GC-MS* Gas Chromatography-Mass Spectrometry, *GEO* Gene Expression Omnibus, *hCG* Human Chorionic Gonadotropin, *HF-LPME* Hollow Fibre Liquid-Phase Microextraction, *HPLC* High-Performance Liquid Chromatography, *IHC* Immunohistochemistry, *LASSO* Least Absolute Shrinkage and Selection Operator, *LC-MS* Liquid Chromatography-Mass Spectrometry, *miRNA* MicroRNA, *MS* Mass Spectrometry, *MS/MS* Tandem Mass Spectrometry, *NGS* Next-Generation Sequencing, *NMR* Nuclear Magnetic Resonance, *NOS* Not Otherwise Specified, *PCR* Polymerase Chain Reaction, *PMB* Postmenopausal Bleeding, *qMSP* Quantitative Methylation-Specific PCR, *qPCR* Quantitative Polymerase Chain Reaction, *qRT-PCR* Quantitative Reverse Transcription Polymerase Chain Reaction, *RIA* Radioimmunoassay, *ROC* Receiver Operating Characteristic, *SELDI-TOF MS* Surface-Enhanced Laser Desorption/Ionisation Time-of-Flight Mass Spectrometry, *SVM* Support Vector Machine, *SWATH-MS* Sequential Window Acquisition of All Theoretical Mass Spectra, *TOF* Time-of-Flight, *UHPLC-QE Orbitrap MS/MS* Ultra-High-Performance Liquid Chromatography Quadrupole Orbitrap Tandem Mass Spectrometry, *UPLC* Ultra-Performance Liquid Chromatography, *UPLC-MS* Ultra-Performance Liquid Chromatography-Mass Spectrometry, *UPLC-QTOF/MS* Ultra-Performance Liquid Chromatography Quadrupole Time-of-Flight Mass Spectrometry, *UV* Ultraviolet, *VAF* Variant Allele Frequency.

Mattila et al. reported elevated urinary epidermal growth factor (EGF) in women with endometrial cancer, consistent with dysregulated EGF-EGFR signalling in epithelial tumorigenesis and detectable prior to treatment [80]. However, subsequent studies showed that urinary EGF levels are influenced by renal synthesis and kidney function, underscoring the need to account for physiological confounders in biomarker models [81, 82]. In contrast, single-enzyme approaches have been less informative. Bazzett et al. found urinary matrix metalloproteinase activity lacked sufficient sensitivity and specificity across gynaecological cancers, highlighting limitations of individual proteases in capturing complex invasive phenotypes [83]. Immune-related proteins have shown greater promise. Urinary neopterin is significantly elevated in endometrial cancer and increased with stage, while serum levels are unchanged, suggesting urine may better reflect tumour-associated macrophage activation and offering potential prognostic value [84]. Urinary CA125 and HE4 have also been shown to be increased in endometrial cancer cases, particularly early stage, compared to symptomatic controls, with accuracy increasing when combined with TVUS (AUC 0.93) [85] (Fig. 2). However, uncertainty remains about performance in asymptomatic or high-risk populations, alongside the nonspecific nature of CA125, which can be elevated in other benign or inflammatory conditions [86].

Discovery-driven proteomics has mapped broader, biologically coherent alterations. Using two-dimensional gel electrophoresis (2-DE), Mu and colleagues reported increased urinary zinc alpha-2 glycoprotein, alpha-1-acid glycoprotein and CD59 and decreased nebulin in people with endometrial cancer, a pattern consistent with immune modulation and cytoskeletal change [87]. 2-DE, however, is not readily scalable for high-throughput clinical testing and translation will require validation in larger cohorts and adaptation to clinically practical platforms. Similarly, applying a discovery-based LC-MS/MS approach, Kacírová et al. identified downregulation of adhesion and basement membrane proteins, including E-cadherin, vitronectin and HSPG2 in urine from endometrial cancer cases compared to controls [88]. While the cohort is small (*n* = 12), these proteomic changes reflect that extracellular matrix biology is mirrored in the urinary proteome [88]. These small studies strengthened the biological rationale for looking beyond single proteins and informed subsequent targeted assays.

Translational studies should prioritise proteins that can be measured reproducibly in clinical laboratories. MCM5, a replication-licensing factor released from proliferating cells, has demonstrated the ability to discriminate endometrial cancer from benign gynaecological conditions, achieving an AUC of 0.83 in a prospective cohort of 41 cases and 58 controls. Measured using a commercial ELISA in full-void urine, this finding supports the feasibility of a clinically tractable assay reflective of tumour-associated proliferative cell shedding into urine [89]. More recently, Njoku et al. applied a quantitative LC-MS workflow integrated with machine learning to urine samples from symptomatic women, identifying a ten-protein panel that reflected processes such as keratinisation, redox balance, complement activation and extracellular matrix biology, achieving an AUC of 0.92 and maintaining performance in early-stage disease. The study illustrates the potential of combining high-resolution proteomics with computational modelling to derive robust, mechanistically informative biomarkers, although translation will require simplification of the pipeline for clinical implementation [34].

Early peptide-based studies demonstrated that low molecular weight urinary gonadotropin (hCG) fragments, particularly the β core fragment, were detectable in 37.8% to 77% of endometrial cancer cases and were more detectable in urine than serum [90, 91]. Although these findings established biological plausibility for hCG-related peptides as urinary indicators of gynaecological malignancy, no subsequent research has validated them as reliable urine biomarkers for endometrial cancer. Glycopeptides enhance biomarker specificity by simultaneously reflecting tumour-associated proteolysis and abnormal glycosylation [92]. Using lectin-enriched fractions with SELDI-TOF, Mu and colleagues identified m/z 1449 as a discriminator for endometrial cancer against ovarian and cervical cancer and m/z 1201 as a separator for ovarian cancer from the other groups, indicating that short urinary molecules can aid differential diagnosis when combined with protein panels [33]. These signals require orthogonal verification and independent cohorts before clinical implementation, given historical concerns about platform reproducibility.

Several practical points will determine the clinical utility of urinary protein or peptide biomarkers. Urine collection technique (e.g., midstream, first void), preservative use, time until centrifugation, storage conditions and how haematuria is managed can all substantially alter protein concentrations and peptide profiles and therefore must be considered [93]. Most reports to date are case control designs and are susceptible to spectrum bias. Prospective, multi centre validation with predefined thresholds, locked algorithms and orthogonal confirmation using clinically relevant targeted MS or immunoassays is needed to develop tests that fit existing pathways for symptomatic women.

## Metabolites as potential biomarkers

Metabolomics captures downstream cellular activity by integrating genomic, transcriptomic and proteomic influences, providing a dynamic, real-time readout of phenotype [94, 95]. In endometrial cancer, metabolic reprogramming reflects key disease drivers, including oestrogen signalling, proliferation and systemic metabolic dysfunction. Because metabolites circulate in biofluids such as urine, metabolomics is well suited to non-invasive cancer detection and monitoring [96]. Metabolomics uses platforms such MS and NMR through two strategies: targeted and untargeted. Targeted approaches measure predefined compounds with high precision [97], while untargeted methods provide a broad metabolic overview and enable novel biomarker discovery, though they require validation [98]. Typically, untargeted studies identify candidates and targeted approaches confirm diagnostic accuracy [96].

Early investigations focused on metabolite classes with known relevance to endometrial cancer, including prostaglandins, steroid hormones and oestrogen derivatives, reflecting the hormone-dependent nature of the disease. Elevated urinary 6-keto-prostaglandin F1α, a marker of inflammatory and angiogenic signalling, has been reported in gynaecological malignancies but is not specific for endometrial cancer detection [99], whereas decreased cortisol, progesterone and androgen metabolite profiles have been observed in people with endometrial cancer [100]. Increased urinary 4-hydroxyestradiol and 16α-hydroxyestrone, biologically active oestrogen metabolites with mitogenic effects on the endometrium, have been observed in both endometrial cancer and hyperplasia, with 16α-hydroxyestrone also increased in people with a higher BMI [101, 102]. Additionally, decreased urinary levels of 2-methoxyestrone and 2-methoxyestradiol have been observed in people with endometrial cancer [101], aligning with the anti-cancer, anti-angiogenic and anti-proliferative actions of these metabolites [103, 104] (Fig. 2). Interestingly, abundance of these metabolites has been shown to be non-discriminatory for endometrial cancer in serum samples [105]. While these findings support the biological plausibility of urine-based metabolic biomarkers, they also illustrate limited disease specificity and the confounding influence of systemic endocrine and metabolic dysregulation.

Untargeted metabolomic approaches aim to comprehensively profile all detectable metabolites, enabling biomarker discovery and pathway analysis. Using LC-MS, Shao et al. identified a five-metabolite urinary panel that could discriminate for endometrial cancer, characterised by decreased porphobilinogen and acetylcysteine and increased N-acetylserine, urocanic acid and isobutyrylglycine [106] (Fig. 2). Diagnostic accuracy of this panel was 83.9%, increasing to 92.9% in a small validation cohort, although hyperplasia was not distinguishable from controls. The implicated metabolites were linked to porphyrin metabolism and oxidative stress responses, processes known to support carcinogenesis [47]. However, the absence of histological and stage information limits clinical interpretation and without independent validation, it remains uncertain whether this panel can reliably support early-stage detection. More broadly, untargeted workflows remain semi-quantitative and susceptible to false discovery, reinforcing the requirement for targeted validation of candidate biomarkers [107].

A substantive advance was reported by Chen et al. who conducted the largest untargeted urinary metabolomic study in endometrial cancer to date (146 endometrial cancer cases and 59 controls), incorporating an internal validation cohort (*n* = 65) and paired serum-urine analyses [108]. Three comparisons were examined: endometrial cancer vs. controls; high-risk vs. low-risk disease; and type I vs. type II endometrial cancer. Thirty-eight differential metabolites were identified in serum and 78 in urine when comparing endometrial cancer cases with healthy controls, with a combined serum-urine panel achieving the highest predictive performance (AUC 0.953 in discovery; 0.947 in validation). Distinct metabolite panels also discriminated high-risk from low-risk disease (AUC 0.881–0.936) and type I from type II cancers (AUC 0.922–0.929). Several discriminatory metabolites were involved in lipid, nucleotide and amino acid metabolism, reflecting the increased biosynthetic demands of proliferating endometrial tumours. Importantly, the urine biomarker panel achieved a higher diagnostic accuracy than serum for discriminating endometrial cancer (AUC 0.947 vs 0.850, respectively). Although disease stage was not specifically evaluated and external validation has not been completed, this study provides strong evidence that urinary metabolites capture clinically relevant aspects of endometrial cancer biology. The same group further demonstrated that urinary metabolic profiles could monitor response to fertility-sparing treatment, supporting potential utility in longitudinal disease surveillance [109].

Pathway-based analyses have provided additional mechanistic insight. Yi et al. applied a pathway-based metabolomic approach, identifying dysregulation of amino acid (tyrosine, arginine, proline, alanine) and nucleotide (purine and pyrimidine) metabolism as central to endometrial cancer biology. Integrated metabolomic and proteomic profiling of endometrial tissue from 44 grade 1-2 endometrioid cancers and 43 controls revealed pathways, which were subsequently validated in urine from an independent cohort, achieving an AUC of 0.808 with the top 100 metabolites. Metabolite changes were directionally discordant across tissue, brushings and urine, highlighting the influence of sampling site, with urine primarily reflecting downstream systemic rather than local tumour metabolism [110]. These findings were independently supported by Fu et al. who examined urine and serum samples from 110 early-stage endometrial cancer cases and 110 controls, confirming enrichment of amino acid metabolic pathways and identifying novel dysregulation of histidine metabolism. High diagnostic performance was achieved using both serum (AUC 0.92) and urinary (AUC 0.97) metabolite panels, with integration of transcriptomic data implicating key metabolic hub genes, including RRM2, TYMS and TK1 [35]. These genes are key regulators of nucleotide synthesis and cell-cycle progression and are consistently overexpressed in endometrial cancer, where their increased expression is associated with enhanced tumour proliferation, genomic instability, adverse clinicopathological features and poor prognosis, underscoring their central role in endometrial cancer development and progression [111–113].

As urinary metabolites arise from both internal physiology and external environmental inputs, many confounding factors must be ruled out before any metabolite can be linked specifically to cancer [96]. Biological variability, including diet, hydration, medications, geographical region and circadian rhythm can significantly influence metabolites. Standardised protocols for sample collection, such as fasting urine and consistent timing, are essential to minimise confounding [114]. More specific to endometrial cancer, metabolic disorders, such as obesity [102] and conditions altering hormones, such as menopause [115], also alter metabolic profiles and should be accounted for in study design. External validation in large, diverse cohorts is critical to ensure reproducibility and diagnostic accuracy before clinical implementation.

## Cytology and single cell technologies

The anatomical continuity of the uterus and lower genital tract means that tumour cells from the endometrium can be detected in urine, especially in people with PMB. Early evidence supporting this concept has largely come from case reports, in which malignant endometrial cells have been identified in urine from women presenting with haematuria or gynaecological symptoms [116–119]. Reported cytological features include marked pleomorphism, papillary architecture and immunophenotypic profiles consistent with Müllerian origin, including PAX8 positivity [119]. Additionally, dual detection of endometrial and bladder malignancies in urine and vaginal samples has been described, highlighting the potential of urine cytology to detect more than one cancer simultaneously [120]. These studies establish biological plausibility but are limited by their reliance on singular case reports. Prospective PMB cohorts are needed to analyse the potential real-world performance.

Building on these observations, O’Flynn et al. conducted a diagnostic accuracy study involving 216 women (103 with known or suspected endometrial cancer and 113 controls with PMB but no cancer). Paired urine and vaginal samples were evaluated by cytology, demonstrating high overall diagnostic performance when both sample types were combined (sensitivity 91.7%, specificity 88.8%). Vaginal cytology showed greater sensitivity for endometrial cancer detection, whereas urine cytology was more specific and achieved superior PPV [39]. Importantly, urine samples were generally adequate for cytological assessment and enabled detection of additional gynaecological and urological malignancies, supporting the feasibility of urine-based cytology within routine clinical workflows. A further large case-control study evaluating the diagnostic accuracy of urogenital cytology samples in women with PMB has completed recruitment [121]; we eagerly await these results.

Despite these promising findings, conventional cytology relies on expert interpretation by specialist cytopathologists, currently limiting its scalability. The integration of digital cytology with machine-learning and AI-based morphological analysis offers the potential to enhance sensitivity, reduce inter-observer variability, automate sample triage and support population-scale screening [122–125]. However, a cytology approach is likely to perform best in women with established or symptomatic disease, where tumour cell shedding is more likely. In contrast, early-stage, low-grade endometrial cancers or atypical hyperplasia cases may exhibit less shedding, limiting sensitivity for detecting these cases, potentially reducing suitability as a stand-alone population screening test [39].

## Conclusion

Endometrial cancer incidence is rising, yet current diagnostic pathways expose many symptomatic women to invasive testing despite a relatively low probability of malignancy. Urine is a uniquely practical biofluid for endometrial cancer biomarker development, capturing both systemic tumour-associated signals and locally shed uterine material, particularly in women presenting with AUB. Across biomarker classes, urinary markers reflect key hallmarks of disease including metabolic reprogramming, immune activation, proliferative cell shedding and genomic instability. At present, urinary DNA methylation and somatic mutation assays show the most diagnostic promise, while cytology performs well in symptomatic cohorts; other domains (proteomics, metabolomics, spectroscopy and miRNAs) provide promising signals but require further standardisation and prospective validation. Standalone urine tests are unlikely to support population screening in the near term; the most realistic application is triage and risk stratification of symptomatic women to reduce unnecessary invasive investigations. Clinical implementation will require prospective validation, standardised workflows and transparent reporting. Ultimately, multimodal urinary biomarker panels integrated with machine learning models into clinical pathways offer the greatest potential to deliver earlier diagnosis, improved patient experience and more efficient use of healthcare resources.
